# Supplementary material for: Electron/infrared-phonon coupling in ABC trilayer graphene
Source: Nat Commun. 2024 Feb 29;15:1888. doi: 10.1038/s41467-024-46129-7 (PMC10904774; doi:10.1038/s41467-024-46129-7)
Supplement: Supplementary file 1 — Supplementary Information [file 41467_2024_46129_MOESM1_ESM.pdf]

Supplementary information for

Electron/infrared-phonon coupling in ABC trilayer graphene

Xiaozhou Zan[1,2†], Xiangdong Guo[3,4†], Aolin Deng[5], Zhiheng Huang[1,2], Le Liu[1,2], Fanfan Wu[1,2], Yalong Yuan[1,2], Jiaojiao Zhao[1,2], Yalin Peng[1,2], Lu Li[1,2], Yangkun Zhang[1,2], Xiuzhen Li[1,2], Jundong Zhu[1,2], Jingwei Dong[1,2], Dongxia Shi[1,2,6], Wei Yang[1,2,6], Xiaoxia Yang[3,4], Zhiwen Shi[5], Luojun Du[1,2*], Qing Dai[3,4*] & Guangyu Zhang[1,2,6*]

[1] *Beijing National Laboratory for Condensed Matter Physics and Institute of Physics, Chinese Academy of Sciences, Beijing 100190, China.*

[2] *School of Physical Sciences, University of Chinese Academy of Sciences, Beijing 100190, China.*

[3] *CAS Key Laboratory of Nanophotonic Materials and Devices, CAS Key Laboratory of Standardization and Measurement for Nanotechnology, CAS Center for Excellence in Nanoscience, National Center for Nanoscience and Technology, Beijing 100190, China.*

[4] *Center of Materials Science and Optoelectronics Engineering, University of Chinese Academy of Sciences, Beijing 100049, China.*

[5] *Key Laboratory of Artificial Structures and Quantum Control (Ministry of Education), Shenyang National Laboratory for Materials Science, School of Physics and Astronomy, Shanghai Jiao Tong University, Shanghai 200240, China.*

[6] *Songshan Lake Materials Laboratory, Dongguan, Guangdong 523808, China.*

[†] *The authors contributed equally to this work.*

*Corresponding authors: luojun.du@iphy.ac.cn; daiq@nanoctr.cn; gyzhang@iphy.ac.cn

As shown in Figure S1, we carried out Raman measurement at 633nm wavelength. The ABC trilayer graphene clearly shows an additional low wave number peak (infrared active phonon mode), which is absent in ABA trilayer graphene (Fig.S1a). Besides, the 2D modes arising from a double-resonant electronic process of ABC and ABA trilayer graphene have almost identical line shapes (Fig.S1b). The Raman spectrum at 633nm wavelength is almost same Raman spectrum with the 532nm wavelength, which indicates the intrinsic characteristics of the sample.

Figure S2-S6 shows the characterization and Raman spectroscopy measurements of the three devices (D1, D2, and D3) we measured. We identified ABC and ABA trilayer graphene through Scanning near field optical microscope (SNOM) in Fig.S2b, Fig.S3b and Fig.S4b. Then we measured the Raman spectra at the corresponding ABC and ABA points, as shown in Fig.S2c-d, Fig.S3c-d and Fig.S4c-d. The transport curve shows that the three devices (D1, D2, and D3) have strong hole doping, and we can detect a charge neutral point (CNP) at 80V in D3, as shown in Fig.S2e, Fig.S3e and Fig.S4e. The Fig.S5-6 shows the Raman shift and Raman spectrum as a function of gate voltage. The Raman Shift is

extracted by Lorentz function fitting. With increasing the hole density, the mode of ABA trilayer graphene and high wavenumber mode of ABC trilayer graphene harden, indicating both are symmetric Raman G mode. In marked contrast, the low wavenumber component of ABC trilayer graphene softens with doping density, confirming the antisymmetric infrared active nature. It is worth noting that the infrared active phonon disappears with hole doping below $|n| = |-10 \times 10^{12}|\ cm^{-2}$, and can only be clearly seen with hole doping above $|n| = |-15 \times 10^{12}|\ cm^{-2}$ in Fig.S6. We also investigated the trilayer graphene device encapsulated in hexagonal boron nitride (h-BN), as shown in Fig.S7. The BN thickness we selected at the top is ~2nm for Snom to identify ABC and ABA trilayer graphene in Fig.S7a. The transport curve shows that the voltage at the CNP is ~0V in Fig.S7b. Then we measure Raman spectra at the corresponding ABC and ABA points (Fig.S7c-d). Fig.S7e-f show the Raman spectrum of phonon G and 2D mode at different gate voltage. At CNP, the ABC and ABA trilayer graphene shows also both a single Raman peak at ~1580 and~1582 cm^{-1}, respectively. Moreover, the 2D modes of ABC and ABA trilayer graphene at CNP have different line shapes. It is almost consistent with the Fig.2b. We did not observe the infrared phonon mode in ABC trilayer graphene when we detected the corresponding carrier density $n \approx \pm 7 \times 10^{12}\ cm^{-2}$ within the gate voltage range of ±100V (CNP at 0V) , which is almost approaching the breakdown limit of the dielectric layer. It is consistent with the above discussion. It is quite surprising that the new infrared active phonon disappears with hole doping below $|n| = |-10 \times 10^{12}|\ cm^{-2}$, and can only be clearly seen with hole doping above $|n| = |-15 \times 10^{12}|\ cm^{-2}$.

Then we investigated the effect of high-temperature annealing (450°C) in Ar/H_2 atmosphere to our graphene samples (S1 and S2) in Fig.S8-9. Fig.S8a-c and Fig.S9a-c show the Raman spectroscopy of ABC and ABA trilayer graphene after annealing (ABC -red line, ABA -blue line) and before annealing (ABC -green line, ABA -black line). Before annealing, the ABC and ABA trilayer graphene shows also both a single Raman peak at ~1580 and~1582 cm^{-1}, respectively. Besides, the 2D modes of ABC and ABA trilayer graphene at CNP have different line shapes. It indicates that the samples are almost in CNP. After annealing, the Raman peaks of ABC and ABA trilayer graphene show a blue shift of 11 cm^{-1} in S1 and 10 cm^{-1} in S2. The ABA trilayer graphene shows a single Raman peak. In striking contrast, ABC trilayer graphene clearly shows an additional low wave number peak, which is absent in ABA trilayer graphene. It is consistent with the previous discussion. It indicates that the graphene samples exhibit strong hole doping after 450°C high temperature annealing in Ar/H_2 atmosphere. And it is due to charge transfer between graphene and SiO_2 surfaces through redox reactions, resulting in hole doping in graphene [1-2].

Fig.S10 shows the SNOM image excited by laser radiation at 1570cm^{-1}, 1572cm^{-1}, 1573cm^{-1}, 1575cm^{-1}, 1578cm^{-1}, 1580cm^{-1}, 1610$cm^{-1}$and 1720cm^{-1}. The ABC trilayer graphene has stronger near-field signal response than ABA trilayer graphene. We have compared the peak features at 10 K and 300 K in Fig.S11. We extracted the Raman Shift and FWHM of phonon G mode of the ABA and ABC trilayer graphene by Lorentz function fitting. We can see that the Raman Shift of phonon G mode of ABA trilayer graphene reduces 2.27 cm^{-1} (0.48 cm^{-1} for ABC trilayer graphene) and the FWHM of phonon G mode of ABA trilayer graphene reduces 0.44 cm^{-1} (0.63 cm^{-1} for ABC trilayer graphene) at 300K compared to 10K. We conclude that as the temperature increases, both the Raman Shift and the FWHM values decrease significantly. Then we carried out circular polarization and linear polarization Raman spectra measurements (Figure S12), and it can be concluded that

phonon G mode and infrared active phonon mode have the same circular polarization response, while the linear polarization response XX and XY are almost identical, indicating that the G peak splitting we observed is not from the stress [3-11] and the boundary [12-14]. At the same time, the supplementary Figure S13 shows that the 1350cm^{-1} D peak of the sample is almost invisible, indicating that the defects of the sample itself are few, or almost no, and the separation of the G peak is not from the defects [15-16]. At the same time, the optical microscope diagram in Fig.1c also shows that the sample is not folded [17-19] and water doped [20], and we also do not carry out chemical ion doping [21-22].

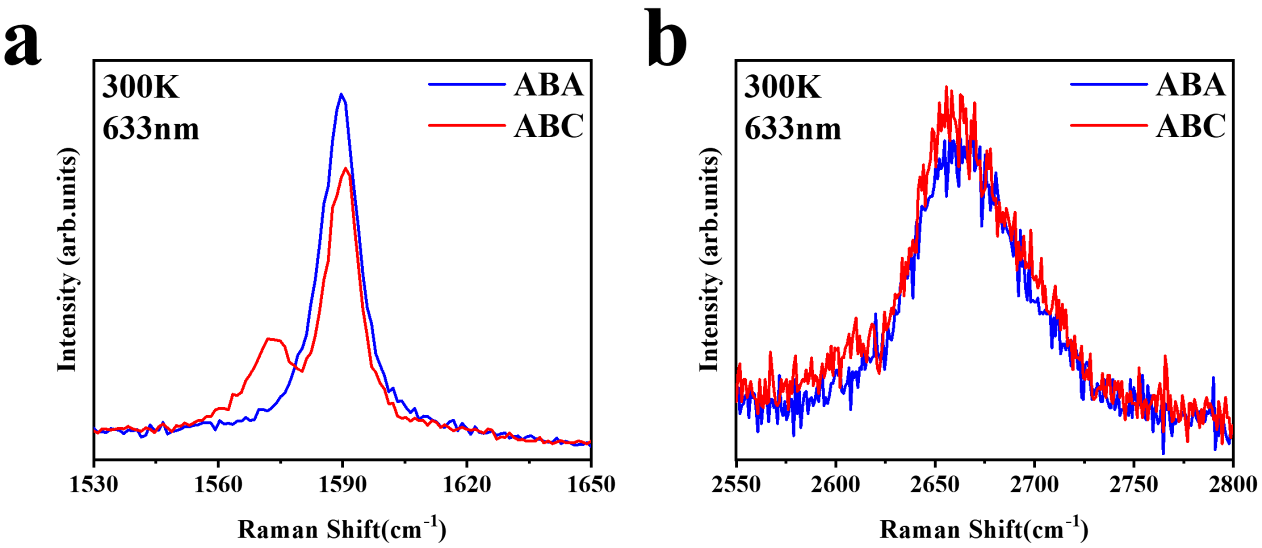

Figure S1. Raman spectrum with excitation wavelength of 633nm.

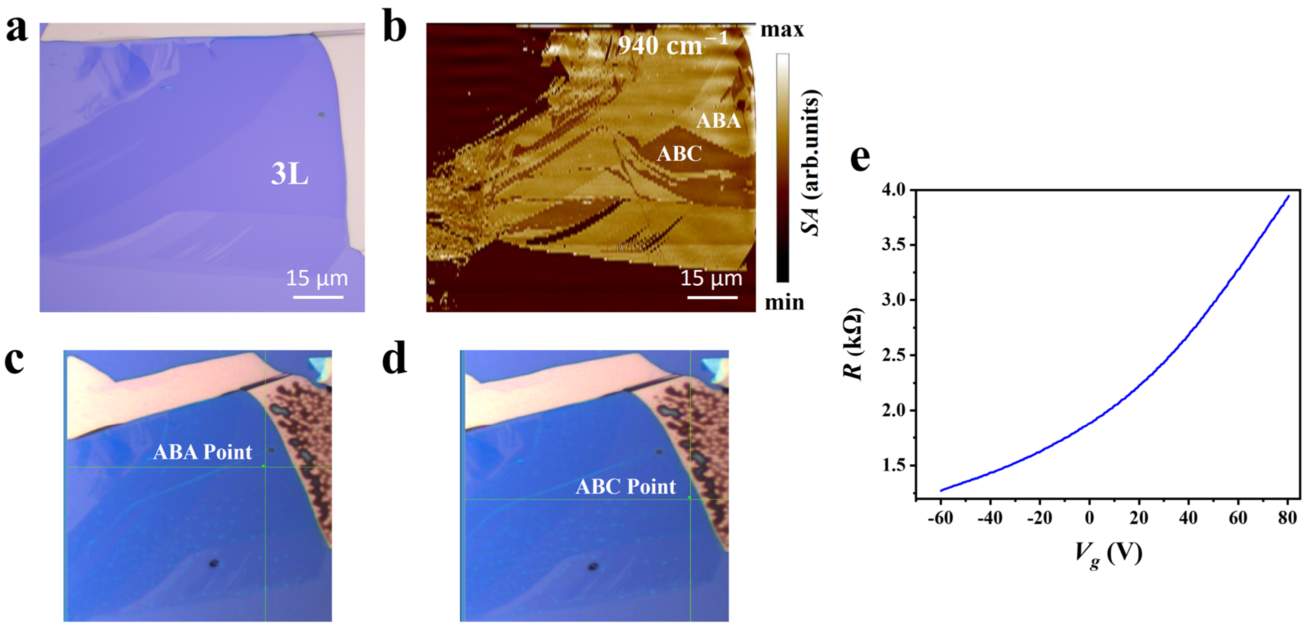

Figure S2. Optical microscope, Snom images and transport curve in graphene Device #1 (D1). a-b Optical microscope and Snom images. The scale bars are 15μm. **c-d** Raman spectrum is measured at ABA and ABC point. **e** transport curve.

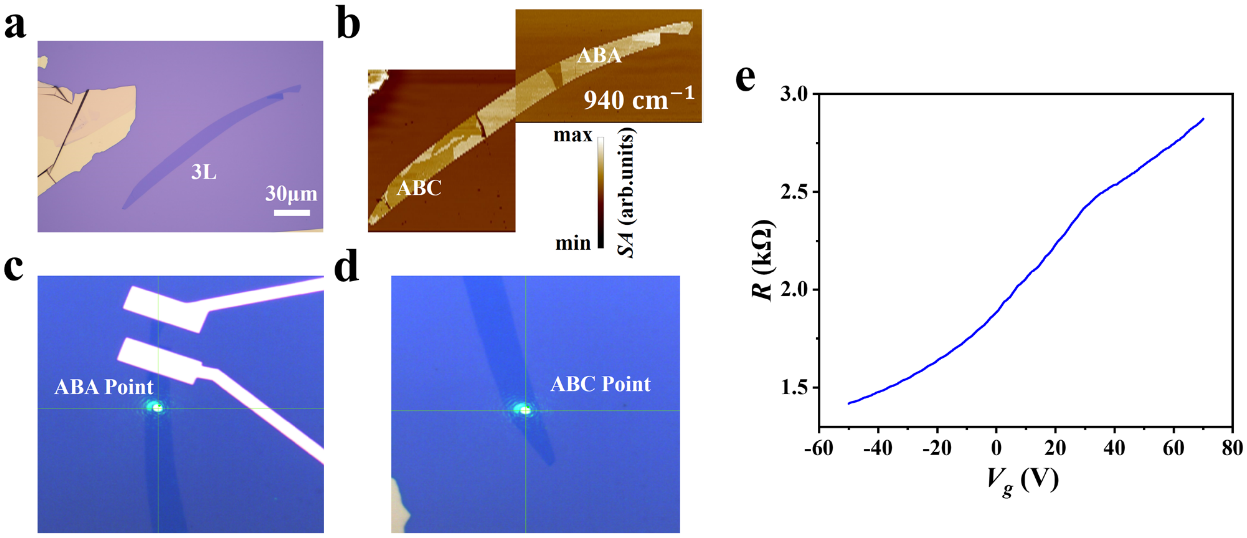

Figure S3. Optical microscope, Snom images and transport curve in graphene Device #2 (D2). a-b Optical microscope and Snom images. The scale bar is 30μm. **c-d** Raman spectrum is measured at ABA and ABC point. **e** transport curve.

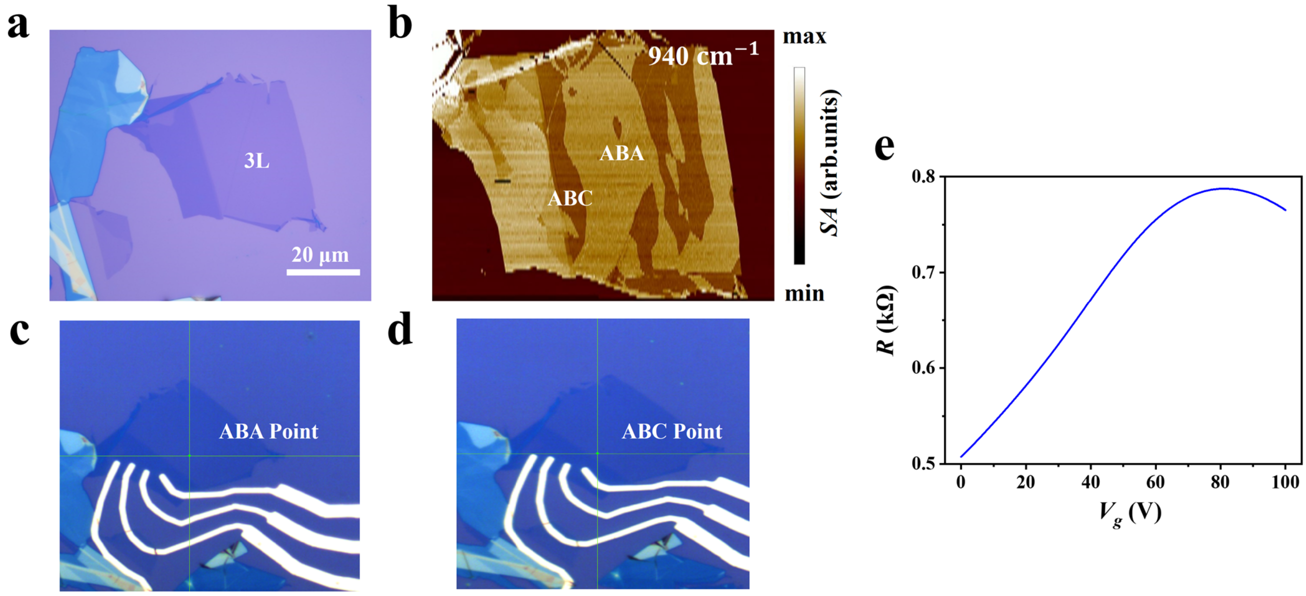

Figure S4. Optical microscope, Snom images and transport curve in graphene Device #3 (D3). a-b Optical microscope and Snom images. The scale bar is 20μm. **c-d** Raman spectrum is measured at ABA and ABC point. **e** transport curve. The CNP voltage of D3 is at 80V.

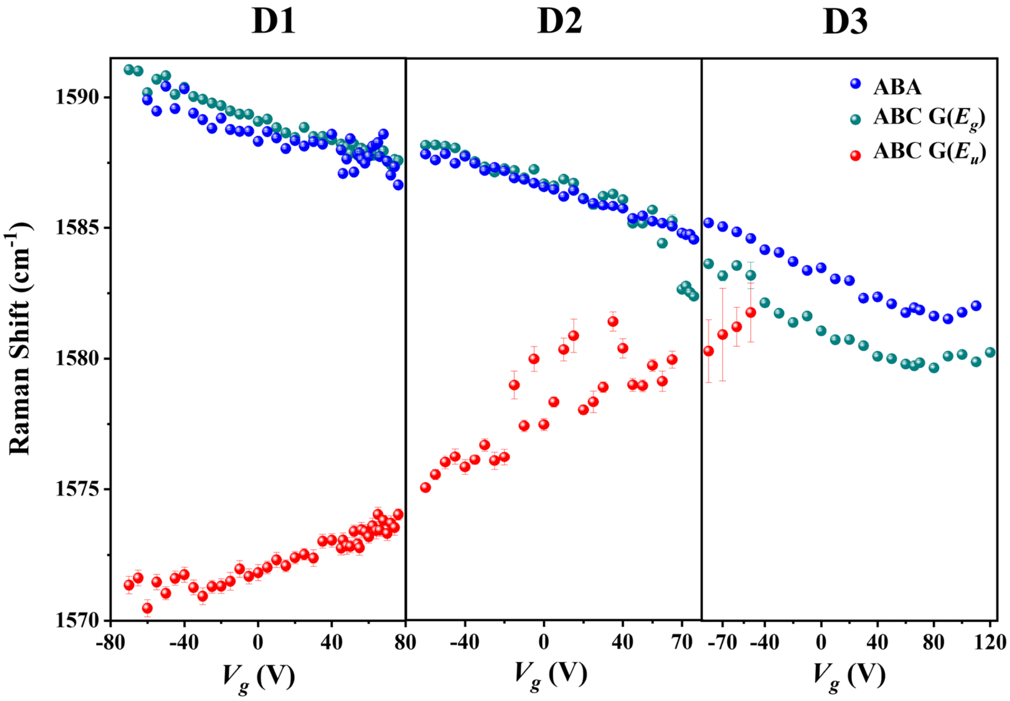

Figure S5. The Raman Shift of phonon G mode of both ABA and ABC trilayer graphene as a function of gate voltages. The Raman Shift is extracted by Lorentz function fitting and the measurements are performed at 10 K. The error bar is the error range obtained by fitting the Lorentz function.

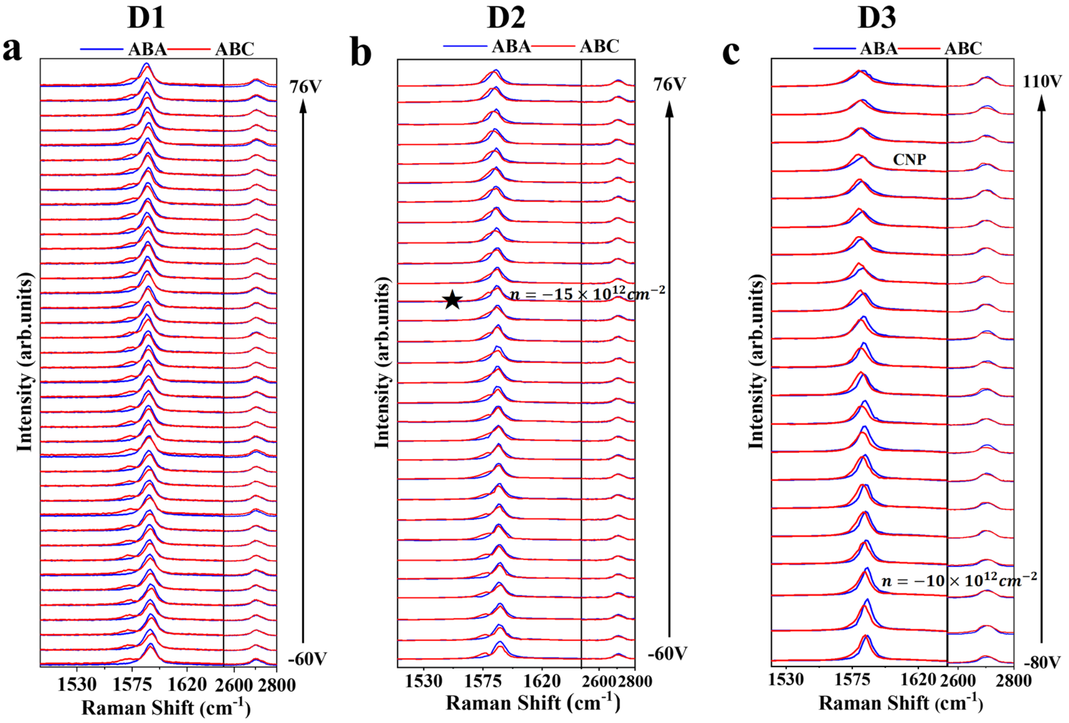

Figure S6. Gate-tunable Raman spectrum of ABC and ABA trilayer graphene in devices (D1, D2 and D3) with excitation wavelength of 532nm at 10K.

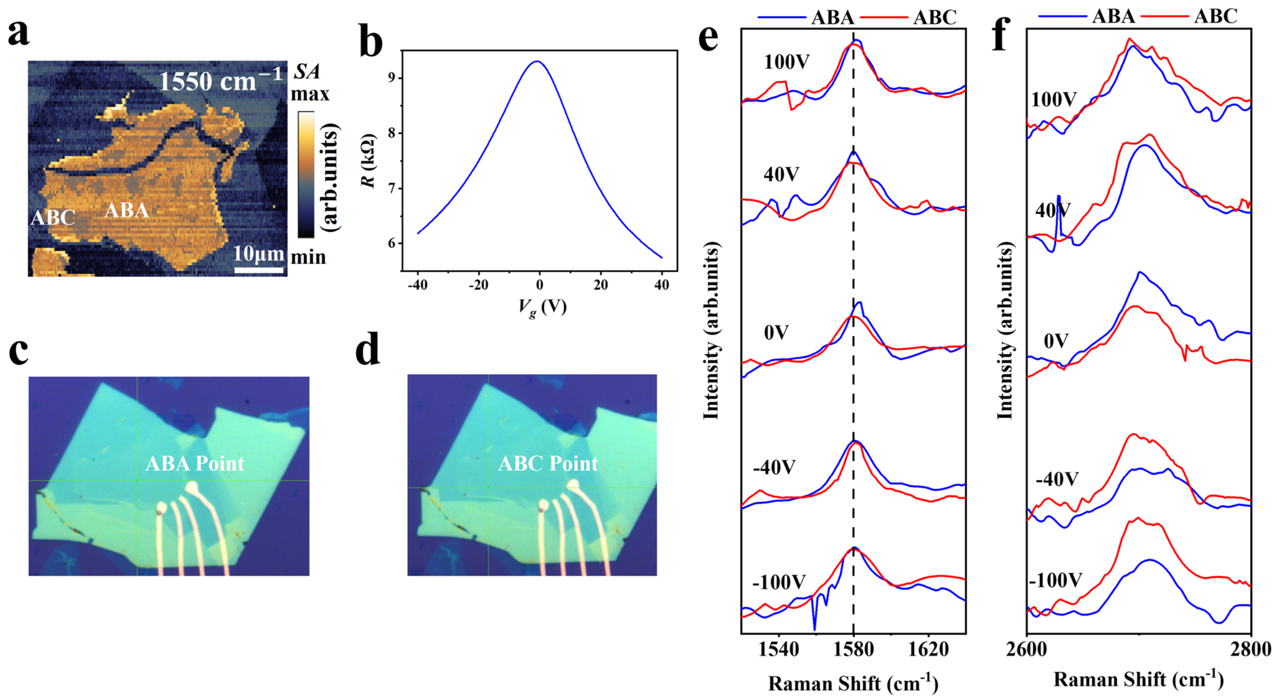

Figure S7. The Raman spectrum and transport curve in h-BN encapsulated trilayer graphene device. **a** The snom nanoimage of h-BN encapsulated trilayer graphene device, the thicknesses of top BN and bottom BN are ~2nm and ~25nm. **b** transport curve. **c-d** Raman spectrum is measured at ABA and ABC point. **e-f** The phonon G and 2D mode of the Raman spectrum at different gate voltages with excitation wavelength of 532nm. The measurement is at 10K.

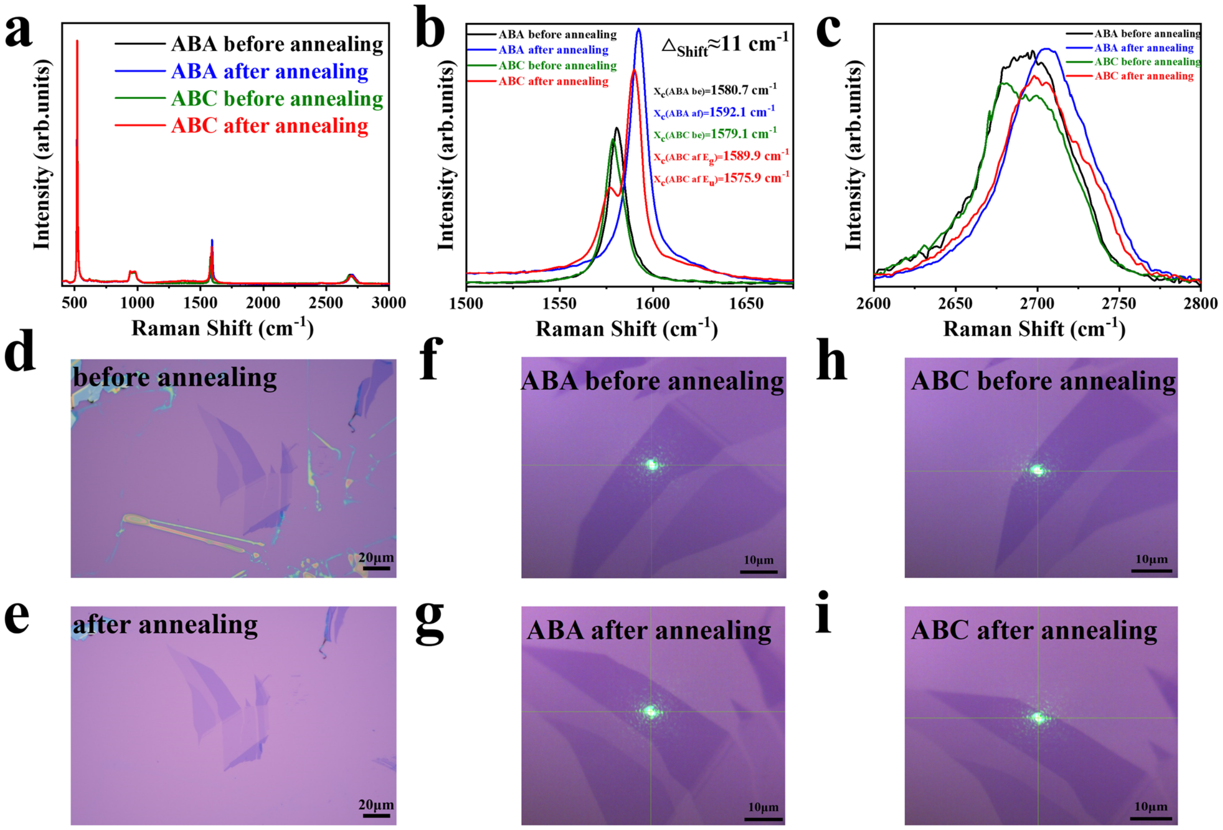

Figure S8. The effect of high temperature 450℃ annealing in Ar/H_2 atmosphere in graphene sample #1 (S1) . a-c The Raman spectroscopy of ABC and ABA trilayer graphene after annealing (ABC -red line, ABA -blue line) and before annealing (ABC -green line, ABA -black line). This measurement is at 300K. **d-i** Optical microscope images of the sample and ABC, ABA points measured by Raman spectroscopy before and after annealing. The scale bars are 20μm and 10μm.

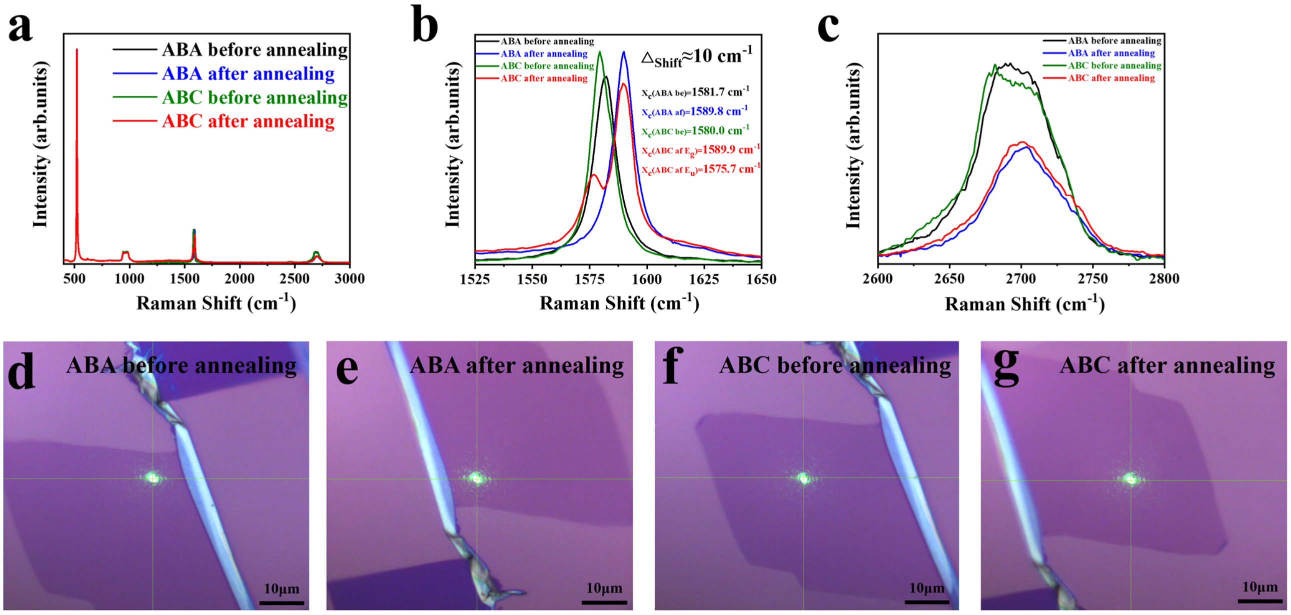

Figure S9. The effect of high temperature 450℃ annealing in Ar/H_2 atmosphere in graphene sample #2 (S2) . a-c The Raman spectroscopy of ABC and ABA trilayer graphene after annealing (ABC -red line, ABA -blue line) and before annealing (ABC -green line, ABA -black line). This measurement is at 300K. **d-i** Optical microscope images of the sample and ABC, ABA points measured by Raman spectroscopy before and after annealing. The scale bars are 10μm.

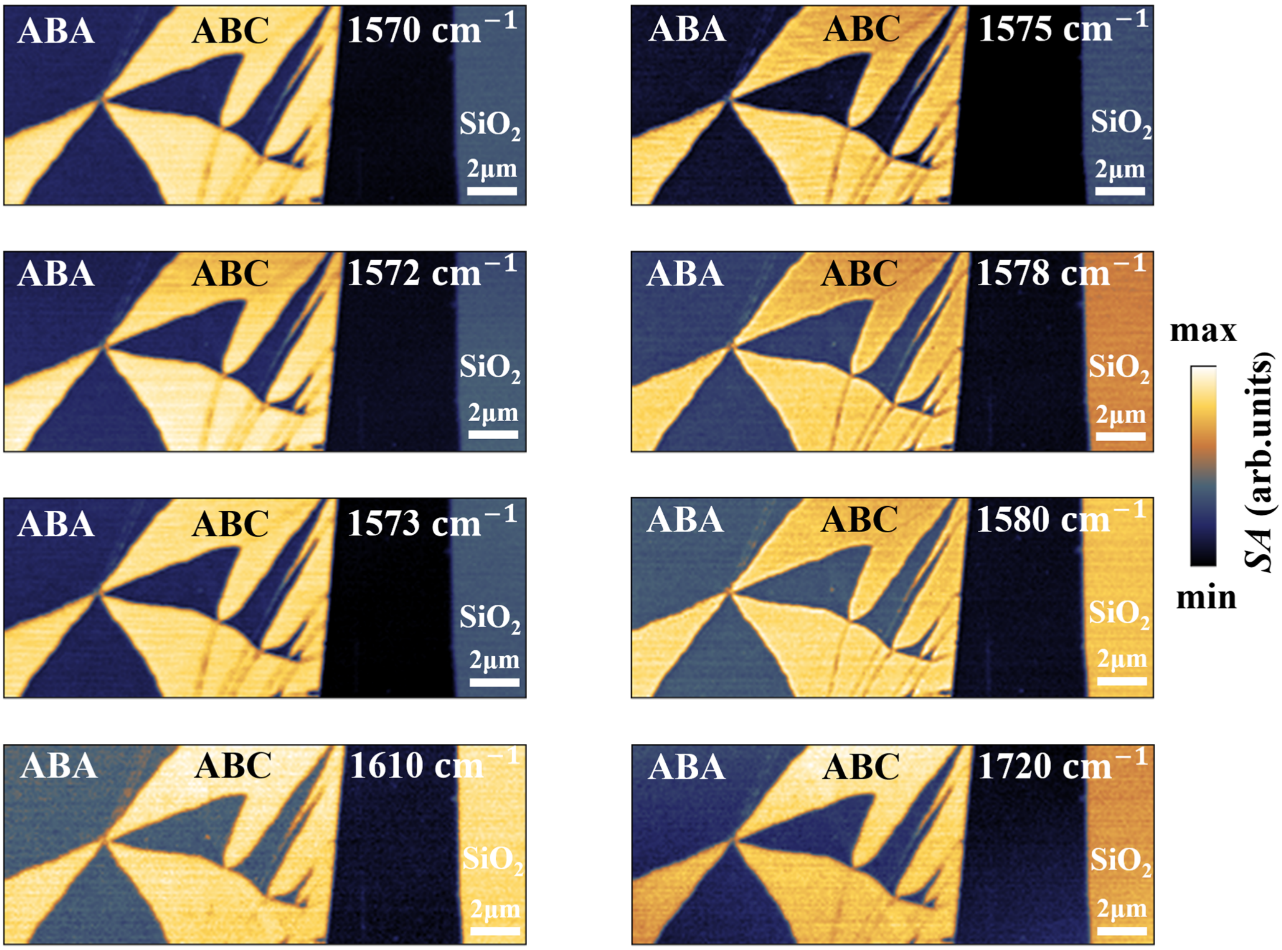

Figure S10. Near-field infrared nanoimages of ABA and ABC trilayer graphene at different excitation frequencies. The scale bars are 2μm.

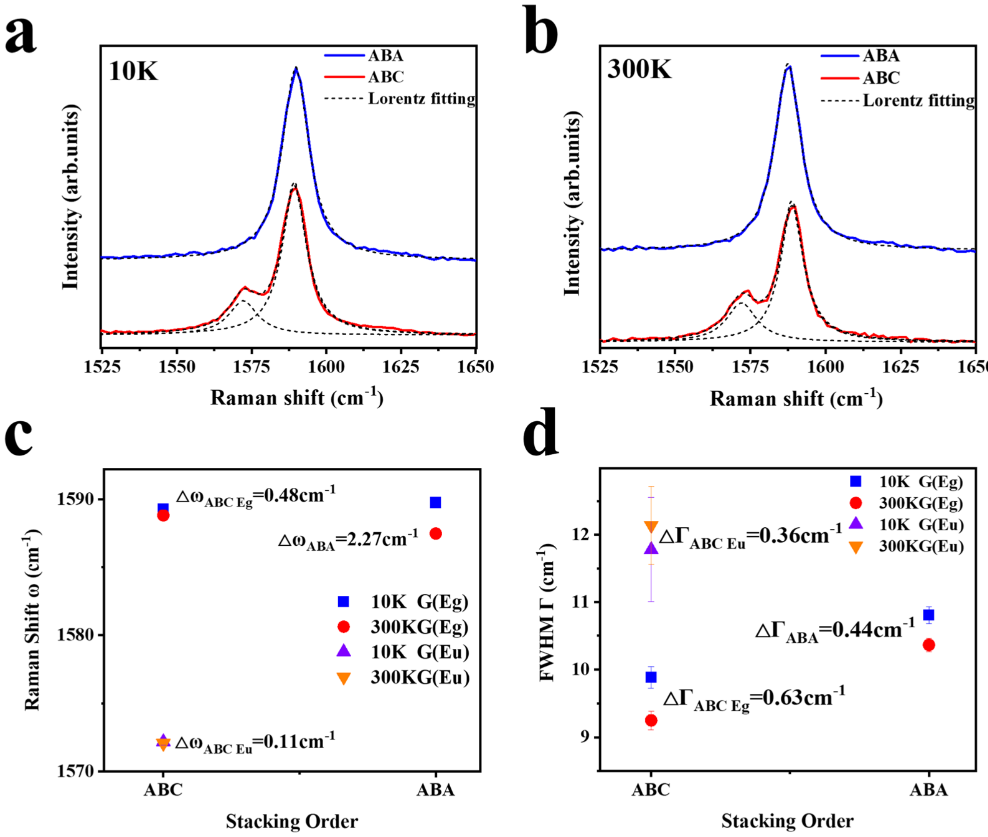

Figure S11. The Raman spectroscopy measurement at 10K and 300K. a-b The Raman spectroscopy of ABC and ABA trilayer graphene at 10K and 300K , respectively, and at the same gate voltage. **c-d** The Raman Shift and full width at half maximum (FWHM) of phonon G mode of both ABA and ABC trilayer graphene is extracted by Lorentz function fitting, respectively. The error bar is the error range obtained by fitting the Lorentz function.

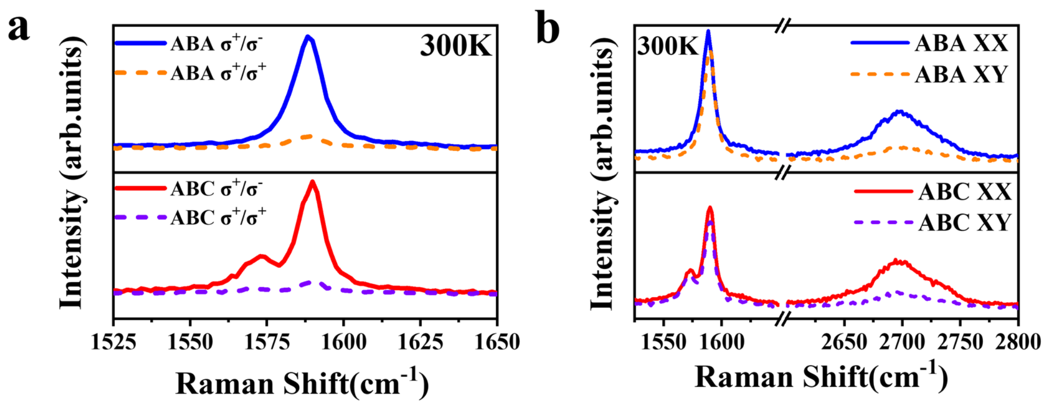

Figure S12. Circularly polarized and linearly polarized Raman spectra. a Circularly polarized Raman spectra. **b** Linearly polarized Raman spectra. They were measured at 300K.

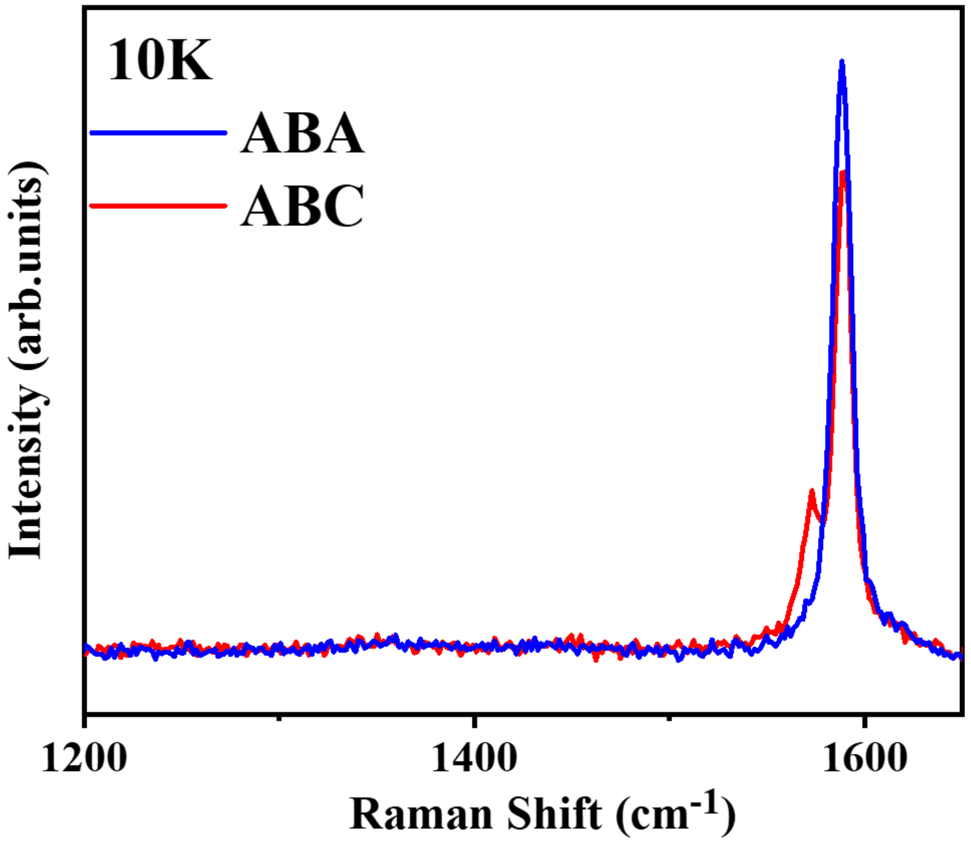

Figure S13. Raman spectrum of 1350 cm^{-1} D peak of trilayer graphene.

Supplementary References

[1] Park. et al. Redox-governed charge doping dictated by interfacial diffusion in two-dimensional materials. Nat. Commun 10, 4931 (2019).

[2] Jahng. et al. Characterizing and controlling infrared phonon anomaly of bilayer graphene in optical-electrical force nanoscopy. Light Sci Appl 12, 281 (2023).

[3] Mohiuddin. et al. Uniaxial strain in graphene by Raman spectroscopy: G peak splitting, Grüneisen parameters, and sample orientation. Phys. Rev. B 79, 205433 (2009)

[4] Mohr. et al. Splitting of the Raman 2D band of graphene subjected to strain. Phys. Rev. B 82, 201409(R) (2010)

[5] Frank. et al. Raman 2D-Band Splitting in Graphene: Theory and Experiment. Acs nano. 5, 2231–2239 (2011).

[6] Yoon. et al. Strain-Dependent Splitting of the Double-Resonance Raman Scattering Band in Graphene. Phys. Rev. Lett. 106, 155502 (2011)

[7] Popov. et al. Theoretical 2D Raman band of strained graphene. Phys. Rev. B 87, 155425 (2013)

[8] Wang. et al. Measuring Interlayer Shear Stress in Bilayer Graphene. Phys. Rev. Lett.119, 036101 (2017)

[9] Huang. et al. Raman Spectral Band Oscillations in Large Graphene Bubbles. Phys. Rev. Lett. 120, 186104 (2018)

[10] Dai. et al. Interface-Governed Deformation of Nanobubbles and Nanotents Formed by Two-Dimensional Materials. Phys. Rev. Lett. 121, 266101 (2018)

[11] Wang. et al. Strain-dependent Raman analysis of the G * band in graphene. Phys. Rev. B 100, 241407(R) (2019)

[12] Yang. et al. Observation of Raman G-Peak Split for Graphene Nanoribbons with Hydrogen-Terminated Zigzag Edges. Nano Lett. 11, 4083–4088 ,(2011)

[13] Cancado. et al. Anisotropy of the Raman Spectra of Nanographite Ribbons. Phys. Rev. Lett. 93,(2004)

[14] Ren. et al. Edge phonon state of mono- and few-layer graphene nanoribbons observed by surface and interference co-enhanced Raman spectroscopy. Phys. Rev. B 81, 035412 (2010)

[15] Eckmann. et al. Probing the Nature of Defects in Graphene by Raman Spectroscopy. Nano Lett. 12, 3925−3930 (2012)

[16] Venezuela. et al. Theory of double-resonant Raman spectra in graphene: Intensity and line shape of defect-induced and two-phonon bands. Phys. Rev. B 84, 035433 (2011)

[17] Poncharal. et al. Effect of rotational stacking faults on the Raman spectra of folded graphene. Phys. Rev. B 79, 195417 (2009)

[18] Podila. et al. Raman Spectroscopy of Folded and Scrolled Graphene. Acs Nano 6, 5784–5790 (2012)

[19] Cong. et al. Evolution of Raman G and G (2D) modes in folded graphene layers. Phys. Rev. B 89, 235430 (2014)

[20] Shim. et al. Water-Gated Charge Doping of Graphene Induced by Mica Substrates. Nano Lett. 12, 648−654 (2012)

[21] Pollak. et al. The Interaction of Li + with Single-Layer and Few-Layer Graphene. Nano Lett. 10, 3386–3388 (2010)

[22] Chaco´n-Torres. et al. Manifestation of Charged and Strained Graphene Layers in the Raman Response of Graphite Intercalation Compounds. Acs Nano 7, 9249–9259 (2013)
